# Documento de consenso para la determinación e informe del perfil lipídico en laboratorios clínicos españoles: ¿Qué parámetros debe incluir un perfil lipídico básico?

**DOI:** 10.1515/almed-2023-0010

**Published:** 2023-05-18

**Authors:** Teresa Arrobas Velilla, Carlos Guijarro, Raquel Campuzano Ruiz, Manuel Rodríguez Piñero, José Francisco Valderrama Marcos, Antonio Pérez Pérez, Manuel Antonio Botana López, Ana Morais López, José Antonio García Donaire, Juan Carlos Obaya, Luis Castilla Guerra, Vicente Pallares Carratalá, Isabel Egocheaga Cabello, Mercedes Salgueira Lazo, María Mar Castellanos Rodrigo, José María Mostaza Prieto, Juan José Gómez Doblas, Antonio Buño Soto, en representación del Grupo Multidisciplinar de Trabajo de Lípidos y Riesgo Vascular

**Affiliations:** Sociedad Española de Arteriosclerosis (SEA), Unidad de Medicina Interna, Hospital Universitario Fundación de Alcorcón, Universidad Rey Juan Carlos, Madrid, España; Sociedad Española de Cardiología (SEC), Unidad de Cardiología, Hospital Universitario Fundación de Alcorcón, Asociación de Riesgo vascular y Rehabilitación Cardiaca de la Sociedad Española de Cardiología, Madrid, España; Sociedad Española de Angiología y Cirugía Vascular (SEACV), Unidad Intercentros Cádiz-Jerez de Angiología y Cirugía Vascular, Hospital Universitario Puerta del Mar, Cádiz, España; Sociedad Española de Cirugía Cardiovascular y Endovascular, Cirugía Cardiovascular (SECCE), Hospital Regional Universitario de Málaga, Málaga, España; Sociedad Española de Endocrinología y Nutrición (SEEN), Sección de Endocrinología, Hospital Universitario Lucus Augusti, Lugo, España; Sociedad Española de Gastroenterología, Hepatología y Nutrición Pediátrica (SEGHNP), Unidad de Nutrición Infantil y Enfermedades Metabólicas, Hospital Universitario La Paz, Madrid, España; Sociedad Española de Hipertensión, Liga Española para la Lucha contra la Hipertensión Arterial (SEH-LELHA), Unidad de Hipertensión Arterial, Hospital Clínico Universitario San Carlos, Madrid, España; Sociedad Española de Medicina de Familia y Comunitaria (SEMFyC), Medicina Familiar y Comunitaria, CS La Chopera,Alcobendas, Madrid, España; Sociedad Española de Medicina Interna (SEMI), Unidad de Hipertensión, Lípidos y Riesgo Vascular, Servicio de Medicina Interna, Sevilla, España; Hospital Virgen Macarena, PCDV Departamento de Medicina, Universidad de Sevilla, Sevilla, España k Sociedad Española de Médicos de Atención Primaria (SEMERGEN), Unidad de Vigilancia de la Salud, Unión de Mutuas, Departamento de Medicina, Universitat Jaume I, Castellón, Castellón, España; Sociedad Española de Médicos Generales y de Familia (SEMG), Medicina Familiar y Comunitaria, Centro de Salud Isla de Oza, Servicio Madrileñõde Salud, Madrid, España; Sociedad Española de Nefrología (SEN), Unidad de Nefrología, Hospital Universitario Virgen Macarena de Sevilla, Sevilla, España; Sociedad Española de Neurología (SEN), Servicio de Neurología Complejo Hospitalario Universitario A Coruña/Instituto de Investigación Biomédica A Coruña, Coruña, España; Sociedad Española de Arteriosclerosis (SEA), Servicio de Medicina Interna, Hospital La Paz-Carlos III, Madrid, España; Sociedad Española de Cardiología (SEC), Servicio de Cardiología, Hospital Universitario Virgen de la Victoria, Málaga, España; Sociedad Española de Medicina de Laboratorio (SEQCML), Servicio de Análisis Clínicos, Hospital Universitario La Paz, Madrid, España; Sociedad Española de Diabetes (SED), Servicio de Endocrinología y Nutrición, Hospital de la Santa Creu i Sant Pau, Barcelona, España

**Keywords:** consenso; panel de lípidos; enfermedades cardiovasculares; bioquímica; colesterol; lípidos; triglicéridos; lipoproteína (a); apolipoproteína B

## Abstract

Las enfermedades cardiovasculares (ECV) siguen siendo la principal causa de muerte en nuestro país. El control adecuado de las alteraciones del metabolismo lipídico es un reto clave en prevención cardiovascular que está lejos de alcanzarse en la práctica clínica real. Existe una gran heterogeneidad en los informes del metabolismo lipídico de los laboratorios clínicos españoles, lo que puede contribuir al mal control del mismo. Por ello, un grupo de trabajo de las principales sociedades científicas implicadas en la atención de los pacientes de riesgo vascular hemos elaborado este documento con una propuesta básica de consenso sobre la determinación del perfil lipídico básico en prevención cardiovascular, recomendaciones para su realización y unificación de criterios para incorporar los objetivos de control lipídico adecuados al riesgo vascular de los pacientes en los informes de laboratorio.

## Introducción

Las enfermedades cardiovasculares (ECV) que incluyen la cardiopatía coronaria y el accidente cerebrovascular, siguen siendo la principal causa de muerte y discapacidad en el mundo [1]. En nuestro país son la primera causa de muerte, por delante de los tumores y de la COVID-19 incluso en el año de su mayor expresión clínica [2]. La arteriosclerosis, como proceso patológico subyacente a la mayoría de las enfermedades cardiovasculares, es una enfermedad que se desarrolla durante décadas y cuyos principales factores de riesgo están bien caracterizados. Uno de los factores de riesgo cuyo tratamiento ha demostrado ser capaz de reducir la morbimortalidad cardiovascular es la dislipidemia [3, 4]. A pesar de disponer de un amplio arsenal terapéutico para el tratamiento de la misma, el grado de control de las alteraciones lipídicas es claramente subóptimo, en especial en los pacientes de riesgo cardiovascular (muy) elevado, en los que la reducción de riesgo absoluto es más importante [5], [6], [7], [8].

Recientemente se han actualizado las Guías Europeas de prevención cardiovascular [9], que son las suscritas por las principales sociedades científicas españolas implicadas en la atención de estos pacientes, incluido el CEIPV (Comité Español Interdisciplinario de Prevención Vascular) [10], [11], [12], [13].

Frente a un amplio consenso sobre los objetivos de tratamiento hipolipemiante ajustado al riesgo vascular, los informes de los laboratorios de bioquímica continúan ofreciendo valores de referencia basados en la distribución de los valores en la población general, eludiendo con frecuencia informar de los valores ‘deseables’ en función del riesgo vascular de los pacientes y grado de ERC (enfermedad renal crónica) vascular de los pacientes. Pese al documento SEA (Sociedad Española de arteriosclerosis)- SEC(Sociedad Española de Cardiología) 2018 [14, 15], muchos informes describen como ‘normales’, valores lipídicos muy por encima de los valores ‘deseables’ en términos de prevención cardiovascular [16] o como ‘anormalmente bajos’ valores lipídicos deseables desde el punto de vista de prevención cardiovascular. Esta información puede ser malinterpretada conduciendo a la abstención terapéutica en pacientes con valores ‘normales’ y la reducción de la intensidad del tratamiento de pacientes con ‘valores anormalmente bajos’. Por ello, un grupo de trabajo de las principales sociedades científicas implicadas en la atención de los pacientes de riesgo vascular, hemos elaborado este documento con una propuesta básica de consenso sobre la determinación del perfil lipídico básico en prevención cardiovascular, recomendaciones para su realización y unificación de criterios para incorporar los objetivos de control lipídico adecuados al riesgo vascular de los pacientes en los informes de laboratorio [17, 18].

## Consideraciones preanaliticas

### ¿Cómo, cuándo y a quién debemos solicitar un perfil lipídico?

La determinación del perfil lipídico es necesaria para conocer el riesgo de presentar enfermedad cardiovascular de la población aparentemente sana o condiciones clínicas de especial riesgo, incluidos los pacientes que van a ser sometidos a cirugía cardiaca. También se requiere para la monitorización de la eficacia terapéutica y la adherencia al tratamiento hipolipemiante. Es imprescindible en prevención cardiovascular especialmente en personas de alto riesgo o con familiares de alto riesgo. Así mismo permite descartar la posible elevación de los parámetros lipídicos secundaria a otras patologías. El grupo de trabajo considera una referencia adecuada las recomendaciones recientes de la Sociedad Europea de Cardiología [9], recientemente traducidas [10] y suscritas por el Comité Español de Prevención Vascular [13] (Tablas 1A and B).

**Tabla 1A:** Determinación de lípidos para valoración de riesgo vascular [19].

**Pacientes sin tratamiento hipolipemiante**

(1). Se recomienda la evaluación de riesgo vascular sistemática completa para personas con cualquier factor de riesgo vascular mayor (p. ej., antecedentes familiares de ECV prematura), hipercolesterolemia familiar (HF), factores de riesgo de ECV como tabaquismo, hipertensión arterial, Diabetes Mellitus (DM), hiperlipemia, ERC, obesidad o comorbilidades que aumenten el riesgo de ECV. (2). Considerar la evaluación sistemática u oportunista del RCV en varones >40 años y mujeres >50 o postmenopaúsicas de la población general sin factores de riesgo. (3). Considerar una reevaluación tras 5 años (o antes si el riesgo se acerca a los umbrales de tratamiento) para todas las personas que hayan pasado un cribado de riesgo de ECV durante un cribado oportunista. (4). No se recomienda la evaluación sistemática del RCV en varones menores de 40 años y mujeres menores de 50 años sin factores de RCV conocidos.

**Monitorización de eficacia terapéutica y adherencia al tratamiento hipolipemiante**

(1). Antes de iniciar el tratamiento con fármacos hipolipemiantes realizar 2 determinaciones separadas 1–2 semanas, excepto tras un evento cardiovascular y en los pacientes con riesgo muy alto con indicación de tratamiento inmediato. (2). Después de iniciar el tratamiento hipolipemiante repetir determinación analítica. Tras evento vascular aterosclerótico agudo, a las 4–6 semanas. En pacientes estables desde el punto de vista cardiovascular a las 8 ± 4 semanas hasta conseguir objetivos. (3). Una vez que el paciente ha alcanzado el objetivo de lípidos óptimo ¿Con qué frecuencia se debe medir los lípidos? Anualmente.

**Tabla 1B:** Objetivos lipídicos según riesgo cardiovascular [9].

En RCV **muy alto** se recomienda una reducción del 50 % del valor basal y un objetivo de c-LDL <1,4 mmol/L (<55 mg/dL), colesterol no HDL <85 mg/dl y ApoB <65 mg/dL. En RCV **alto**, se recomienda una reducción del 50 % del valor basal y un objetivo de c-LDL<1,8 mmol/L (<70 mg/dL), colesterol no HDL <100 mg/dL y ApoB <80 mg/dL. En RCV **moderado**, se debe considerar un objetivo de c-LDL < 2,6 mmol/L (<100 mg/dL) colesterol no HDL <131 mg/dl y ApoB <100 mg/dL. En RCV **bajo**, se puede considerar un objetivo de c-LDL < 3,0 mmol/L (<116 mg/dL).
**Sospechar hipercolesterolemia familiar** en pacientes que tengan enfermedad cardiovascular arteriosclerótica antes de los 55 años (varones) o de los 60 (mujeres), en personas con un familiar que haya tenido ECV prematura, quienes tengan familiares con xantomas tendinosos, pacientes con c-LDL muy aumentado (adultos, >5 mmol/L [190 mg/dL]; niños, > 4 mmol/L [150 mg/dL]) y familiares de primer grado de pacientes con hipercolesterolemia familiar.
Para los niños, se recomienda realizar las pruebas **desde los 5 años o antes** cuando se sospeche hipercolesterolemia familiar homocigota (HFHo).

### Factores que influyen en la determinación del perfil lipídico del paciente

Múltiples factores pueden influir en los parámetros analíticos; preferiblemente, la toma de muestra debe realizarse en un estado “metabólicamente estable” [20].

**Table:** 

Recomendación 1: Se desaconseja la determinación de niveles lipídicos en el contexto de un proceso inflamatorio agudo no cardiovascular. Se recomienda la determinación de niveles lipídicos en las primeras 24 h de un proceso isquémico agudo arterioscleroso.

#### Estilo de vida y condiciones fisiopatológicas del paciente:

(a) Deben mantenerse hábitos estables las dos semanas previas a la extracción.
(b) No realizar ejercicio físico extremo previo a la extracción.
(c) Permanecer sentado 15 minutos previos a la extracción.
(d) Se recomienda para la estandarización de la flebotomía: Obtención de la sangre venosa con el paciente en posición sentada, (puede existir concentración más baja de CT y c-LDL en posición supina).
(e) Descartar dislipidemias secundarias y asociadas a tratamiento farmacológico (Material Suplementario, Anexo 1 [21, 22]).
(f) Esperar un mínimo de 2–4 semanas tras un proceso inflamatorio agudo pues pueden provocar descenso del colesterol total, del colesterol LDL, del colesterol HDL y un incremento de triglicéridos [23–26].
(g) Se recomienda una determinación de parámetros lipídicos tras síndrome coronario agudo (u otro proceso isquémico agudo) en las primeras 24 h [27–29]. Si se realiza >24 h después del proceso agudo, debe tenerse en cuenta en la toma de decisiones que los niveles de colesterol total y LDL pueden estar disminuidos en relación con los habituales del paciente. Se recomienda la determinación de los niveles de Lp (a) en los pacientes en los que no se haya determinado previamente. Aunque los niveles de Lp (a) pueden elevarse en el contexto de un proceso agudo, la variación es modesta [30, 31], lo que permite detectar a pacientes con Lp (a) marcadamente elevada en fase precoz.

#### ¿Es necesario el ayuno para el análisis del perfil lipídico?

– La mayor parte de las determinaciones lipídicas ofrecen resultados similares independientemente de la situación de ayuno del paciente [32].
– Las principales guías clínicas no exigen ayuno al menos para una evaluación inicial del riesgo o para diagnosticar una hipercolesterolemia aislada como Hipercolesterolemia familiar o Lp(a) elevada sin elevación concomitante de triglicéridos. Los lípidos sin ayunas pueden predecir mejor el riesgo de ECVA ya que reflejan mejor el estado postprandial del paciente y la influencia del riesgo residual [33].
– La concentración de triglicéridos es la única magnitud que cambia significativamente tras la ingesta [33]. Dada la inexactitud de la ecuación de Friedewald en pacientes con Tg>150 mg/dL es preferible realizar la estimación de c-LDL por la fórmula de Martin/Hopkins [34] (Material Suplementario Tabla S3) o utilizar el c-No HDL en estos pacientes.
– Se recomienda ayuno si Tg≥4,5 mmol/L (≥398 mg/dL), antes de iniciar tratamientos farmacológicos que pueden causar hipertrigliceridemia grave (p. ej., isotretinoína), en individuos genéticamente predispuestos, con historia de pancreatitis hipertrigliceridémica y cuando se realicen pruebas de laboratorio adicionales que requieran muestras en ayunas o matutinas (p. ej. glucosa en ayunas o marcadores con ritmo circadiano).
– Las mediciones de los perfiles de lípidos en ayunas y no ayunas deben considerarse complementarias y no mutuamente excluyentes.
– Las determinaciones de colesterol y triglicéridos se realizan de modo habitual mediante métodos enzimáticos con una variabilidad de las determinaciones inferior al 10 % (Material Suplementario Tabla S2) [18]. No obstante, debido a la variabilidad biológica intraindividual y en las condiciones de recolección de la muestra de los parámetros lipídicos (≈20 % para triglicéridos y≈10 % para c-HDL y c-LDL) es razonable realizar una segunda determinación de parámetros lipídicos en los pacientes en prevención primaria que no tengan una indicación claramente establecida para iniciar tratamiento hipolipemiante sin demora [18].

**Table:** 

Recomendación 2: No se requiere de rutina ayuno para la determinación de un perfil de lípidos en la valoración del riesgo inicial. Si la concentración de triglicéridos es superior a Tg≥4,5 mmol/L (≥398 mg/dL), es recomendable una segunda determinación en ayunas para confirmación.

## Consideraciones analíticas

### ¿Se debe informar de la metodología analítica?

La cuantificación de parámetros lipídicos debe realizarse siempre con la misma metodología. En caso de producirse un cambio, éste debe ser notificado. El conocimiento del método analítico empleado para la determinación de parámetros lipídicos es necesario, ya que pueden existir diferentes interferencias o interpretaciones erróneas.

**Table:** 

Recomendación 3: Informar de la metodología de las técnicas analíticas o modificación de unidades es esencial para una correcta interpretación de los resultados de laboratorio.

### Métodos para determinar el c-LDL

El método de referencia para la determinación de c-LDL es la separación de lipoproteínas en gradiente de densidad por ultracentrifugación, una técnica tediosa y sólo disponible en laboratorios especializados. Por este motivo, tradicionalmente se realiza una estimación a partir de la medición del colesterol y triglicéridos totales (por métodos enzimáticos) y la determinación directa del colesterol HDL. La fórmula de Friedewald es la utilizada con más frecuencia [35].

Fórmula de Friedewald para la estimación del Colesterol LDL (en mg/dL):

Colesterol LDL=Colesterol total−Colesterol HDL – Triglicéridos/ 5

La fórmula de Friedewald asume la ausencia de quilomicrones y una proporción fija de colesterol/Tg en las VLDL (1/5 en mg/dL; 1/2.2 en mmol/L). Debido a que la relación Tg/Colesterol en las VLDL aumenta progresivamente a medida que la hipertrigliceridemia se acentúa, la ecuación sobrestima el colesterol de las VLDL y por tanto, subestima el c-LDL en pacientes hipertrigliceridémicos. La ecuación presenta una exactitud aceptable cuando la concentración Tg es<200 mg/dL y a partir de TG>400 mg/dL no debe utilizarse. (Recomendación 4).

La ecuación de Martin-Hopkins reemplaza el número 5 de la estimación de Friedewald (c-VLDL=Tg/5) por divisores que varían según los valores de Tg y c-no HDL del paciente (Material Suplementario Tabla S3) [34]. La ecuación de Martin-Hopkins muestra una mejor precisión que la de Friedewald para Tg>150 mg/dL, para niveles de c-LDL <100 mg/dL, y especialmente para <70 mg/dL.

La fórmula de Sampson es más compleja y presenta resultados similares a la de Martin- Hopkins para pacientes con Tg<400 mg/dL, por lo que es de uso menos frecuente. En los pacientes con Tg>400 mg/dL no es recomendable el uso de fórmulas para la estimación del c-LDL por su menor fiabilidad.

La ultracentrifugación, método clásico de referencia para la determinación de c-LDL, es un método laborioso que sólo se emplea en laboratorios muy especializados. Disponemos de un método directo para su cuantificación, preciso y ampliamente disponible en muchos laboratorios. La implementación de este marcador se recomienda cuando los triglicéridos son superiores a 400 mg/dL o LDL <70 mg/dL, situación en la cual las fórmulas de estimación del c-LDL son más inexactas [34].

Si no se dispone de determinación directa de c-LDL se recomienda el uso del c no-HDL como marcador del colesterol ‘aterogénico’ [36] o la determinación de apolipoproteina B (ApoB). El c no-HDL no requiere la determinación de Tg, no se ve influido por el ayuno y tiene una alta correlación con los niveles de ApoB.

**Table:** 

Recomendación 4: La ecuación de Friedewald es precisa en la mayoría de los pacientes con c-LDL >100 mg/dL y Tg<150 mg/dL. La ecuación modificada de Martin-Hopkins es preferible para el cálculo de c-LDL, sobre todo en pacientes con concentraciones bajas de LDLC <70 mg/dL, concentraciones de Tg de 150–400 mg/dL y en muestras sin ayuno. Los ensayos de c-LDL directo deben usarse para la evaluación de c-LDL cuando la concentración de Tg es ≥400 mg/dL.

En pacientes con elevación importante de Lp(a), la estimación de c-LDL debe corregirse con la fórmula:

c-LDL corregido por Lp(a) (mg/dL)=c-LDL (mg/dL)−[Lp (a) (mg/dL)×0,30]

c-LDL corregido por Lp(a) (nmol/L)=c−LDL (nmol/L)−[Lp (a) (mg/dL)×0,0078]

La posible elevación de Lp(a) debe tenerse en consideración en especial en pacientes subsaharianos, pacientes con síndrome nefrótico, en diálisis peritoneal o con un descenso de c-LDL deficiente tras recibir tratamiento hipolipemiante.

## Consideraciones postanalíticas

### Marcadores de ‘normalidad’ y alertas

El laboratorio clínico es clave para la estimación del riesgo cardiovascular de los pacientes con dislipidemia. Es de vital importancia establecer valores de referencia diferenciados para la población pediátrica.

Es deseable que las determinaciones lipídicas se referencien a los valores deseables en términos de riesgo y prevención cardiovascular [14], [15], [16]. En la Tabla 2, se muestran los valores deseables de los principales parámetros lipídicos de las sociedades Europeas de Cardiología, Arteriosclerosis y Medicina de Laboratorio (España) 2019 [17–19] para adultos.

**Tabla 2:** Valores lipídicos deseables adultos según las sociedades Europeas de Cardiología, Arteriosclerosis y Medicina de Laboratorio [17–19].

Parámetro	Valor deseable adultos
Colesterol total	<200 mg/dL (5,17 mmol/L)
Colesterol-HDL	>50 mg/dL Mujeres (>1,29 mmol/L)
	>40 mg/dL Hombres (1,03 mmol/L)
Colesterol No- HDL	Valores recomendados según el RCV
	– Prevención secundaria y RCV Muy alto <85 mg/dL (<2,2 mmol/L)
	– RCV Alto <100 mg/dL; (<2,6 mmol/L)
	– RCV Moderado <130 mg/dL (<3,4 mmol/L)
Colesterol LDL	Valores recomendados según RCV
	– Prevención secundaria y RCV Muy alto <55 mg/dL (<1,4 mmol/L)
	– RCV Alto <70 mg/dL (<1,8 mmol/L)
	– RCV Moderado <100 mg/dL (<2,6 mmol/L)
	– RCV Bajo <116 mg/dL (<3 mmol/L)
Colesterol de partículas residuales	<30 mg/dL (0,78 mmol/L) en ayunas < 35 mg/dL (0,91 mmol/L) no enayunas
Triglicíridos	TG <150 mg/dL en ayunas (<1,69 mmol/L)
	(TG <175 mg/dL no en ayunas) (<1,97 mmol/L)
Apolipoproteína B	Valores recomendados según RCV
	– Prevención secundaria y RCV Muy alto <65 mg/dL (<1,27 mmol/L)
	– RCV Alto <80 mg/dL (<1,56 mmol/L)
	– RCV Moderado <100 mg/dL (<1,95 mmol/L)
Lp (a)	<50 mg/dL (<105 nmol/L)

RCV, riesgo cardiovascular; Lp (a), lipoproteína (a) HDL, lipoproteínas de alta densidad; LDL, lipoproteínas de baja densidad. Colesterol de partículas residuales = CT–c-LDL–c-HDL.

Aquellos valores que puedan ser considerados como “críticos” deben incorporar una alerta al médico peticionario, como se muestra en la Tabla 3.

**Tabla 3:** Alertas recomendadas para el Sistema informático/Informe de Laboratorio.

Parámetro	Valor crítico	Alerta
Colesterol total	310 mg/dL	Paciente de alto riesgo vascular
Triglicéridos	TG>880 mg/dL	Hipertrigliceridemia severa con riesgo de pancreatitis aguda
Colesterol LDL adultos	>190 mg/dL	Considerar hipercolesterolemia familiar heterocigota
Colesterol LDL adultos	>500 mg/dL	Considerar hipercolesterolemia familiar homocigota.
Triada lipídica aterogénica	Si: TG>150 mg/dL y c-HDL<30 mg/dL c-LDL/ApoB<1,3 o Tg/c-HDL>2	Triada lipídica orientativa de dislipidemia aterogénica de muy alto riesgo vascular
Lipoproteína (a)	>120 mg/dL (260 nmol/L)^a^	Riesgo muy elevado de enfermedad cardiovascular aterosclerosa y estenosis de la válvula aórtica
Apolipoproteína A 1	<10 mg/dL	Valorar Hipoalfalipoproteinemia
Apolipoproteína B	<10 mg/dL	Valorar Hypobetalipoproteinemia

^a^Estimacion según document consenso EAS /EFLM.

### ¿Qué parámetros tiene que incluirse en un perfil lipídico básico?

**Figura 1:**
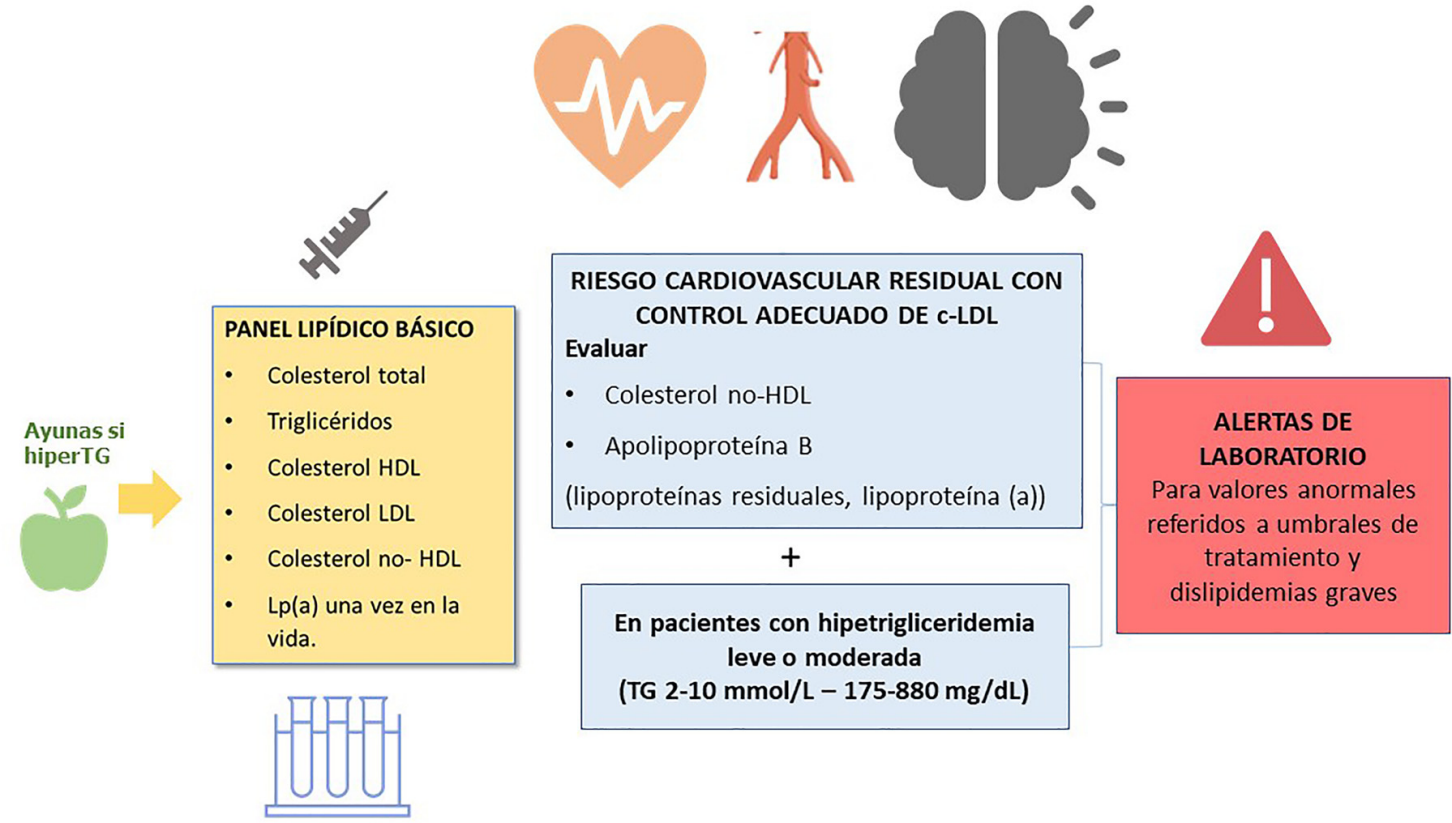
Recomendaciones básicas para el informe de perfil lipídico en laboratorios clínicos españoles.

El perfil lipídico básico debe incluir la determinación de colesterol total, colesterol HDL, triglicéridos, colesterol no-HDL y colesterol LDL [9, 19, 37–39] (Figura 1).

**Table:** 

Recomendación 5: Los valores de referencia de los parámetros lipídicos en los informes de laboratorio siempre deben referirse al riesgo del paciente y no a valores de normalidad poblacionales.La presencia de asteriscos en valores fuera del intervalo de normalidad poblacional es desaconsejado. Se recomiendan sistemas de alerta para niveles lipídicos extremos sugerentes de dislipidemias graves. Se deben establecer valores específicos para población pediátrica.

Los documentos de consenso de la Sociedad Europea de Arteriosclerosis y la Sociedad Europea de Medicina de Laboratorio recomiendan también la estimación de partículas remanentes [9], [10], [11], [12], [13], [14], [15], [16], [17]. La lipoproteína (a) elevada confiere un aumento del riesgo vascular, por lo que su determinación es también aconsejable al menos una vez en la vida del paciente dado que los niveles están determinados sustancialmente por la genética [9], [10], [11], [12], [13], [14], [15], [16], [17].

En los pacientes con Tg>400 mg/dL es recomendable la determinación directa del c-LDL para obtener cifras más fiables [40]. Si está disponible, la determinación de ApoB es un marcador de especial interés, puesto que es el mejor marcador del número de lipoproteínas aterogénicas [41]. Si la determinación directa de c-LDL o ApoB no están disponibles, puede usarse como aproximación el colesterol no HDL.

**Table:** 

Recomendación 6: El perfil lipídico básico ha de consistir en la determinación de colesterol total, colesterol HDL, triglicéridos, c no-HDL y estimación del c-LDL.La lipoproteína (a) debería evaluarse al menos una vez en la vida. En los pacientes con hipertrigliceridemia leve o moderada es recomendable la determinación de colesterol no-HDL y ApoB para la valoración del riesgo cardiovascular residual.

### ¿ Cual es la utilidad de determinar colesterol no HDL?

La estimación del colesterol no HDL, es un cálculo sencillo (colesterol total – c-HDL), representa el colesterol de las lipoproteínas aterogénicas y tiene una elevada correlación con los niveles de ApoB. Es el parámetro lipídico de referencia para la estimación del riesgo vascular con las ecuaciones SCORE2 (Systematic Coronary Risk Evaluation) y SCOREOP (*Systematic Coronary Risk Evaluation old people*) [9, 42, 43]. Una ventaja adicional es que no está afectado por el ayuno, puede determinarse en pacientes con concentración de Tg>400 mg/dL o servir de orientación en laboratorios que no dispongan de determinación de LDL directo o ApoB [44].

### ¿Cuándo usar apolipoproteína B?

La ApoB Es un excelente predictor de eventos cardiovasculares ya que esta apoproteína está presente en las principales lipoproteínas aterogénicas: LDL, lipoproteína a, VLDL e IDL [41], [42], [43], [44], [45]. La medición de ApoB es equivalente a cuantificar el número de lipoproteínas aterogénicas, ya que cada una de ellas contiene una única molécula de ApoB. Los valores de ApoB no varían por la situación de ayuno. El número de lipopartículas puede también medirse mediante RNM (resonancia magnética nuclear), pero esta técnica no está disponible en práctica clínica asistencial [46].

La ApoB tiene un valor especial en personas con triglicéridos elevados, diabetes mellitus, obesidad, Síndrome metabólico o c-LDL muy bajo, en los que la medición o estimación de colesterol LDL puede ser inexacta, además de no recoger el componente aterogénico de otras lipoproteínas.

La determinación de ApoB no suele formar parte del perfil lipídico estándar y de los modelos de estimación del riesgo de ECVA (enfermedad cardiovascular aterosclerosa). Los trastornos monogénicos como la hipercolesterolemia familiar (HF) puede reconocer fácilmente a partir del panel de lípidos estándar sin necesidad de medir la ApoB (Material Suplementario Tabla S4) [47]. Por otro lado, la concentración de ApoB puede ayudar en la tipificación de dislipidemias más severas como la hiperlipidemia familiar combinada y la disbetalipoproteinemia familiar [48] (Material Suplementario Tabla S6).

**Table:** 

Recomendación 7: Se recomienda la determinación de ApoB para la evaluación de riesgo vascular, tipificación de dislipidemias, caracterización del tamaño de partículas, y puede preferirse a colesterol no HDL, en personas con hipertrigliceridemia leve a moderada (175–880 mg/dL),diabetes, obesidad, síndrome metabólico, o c-LDL muy bajo (<70 mg/dL).

### ¿Cuándo determinar lipoproteína (a)?

Se recomienda determinar Lp (a) al menos una vez en la vida para estimación de riesgo vascular [9, 49], [50], [51], [52]. Esta determinación es especialmente relevante en pacientes con enfermedad cardiovascular prematura, hipercolesterolemia familiar, pobre respuesta al tratamiento con estatinas, estenosis aórtica o eventos isquémicos recurrentes y obviamente los familiares de pacientes con Lp (a) elevada. Los pacientes con Lp (a) muy elevada (>180 mg/dL/>430 nmol/L) tienen un riesgo cardiovascular equivalente a la de los pacientes con hipercolesterolemia familiar heterocigota [53, 54]. Uno de los problemas de la medición de Lp (a) es la variabilidad de resultados con distintas técnicas de detección y la ausencia de una equivalencia directa entre los niveles reportados en mg/dL y nmol/L según las distintas isoformas de apoproteína (a).

La determinación de Lp (a), debido a su marcada influencia genética y falta de tratamientos farmacológicos específicos, debe determinarse únicamente una vez en la vida. Las excepciones a esta regla son la transición a la menopausia, el embarazo, el uso de anticonceptivos orales, la enfermedad renal crónica o síndrome nefrótico o cuando se administre un tratamiento específico para reducir la Lp (a) o para modular las opciones terapéuticas aconsejables, como el uso de inhibidores de PCSK9 [55].

**Table:** 

Recomendación 8: Determinación de lipoproteína a una vez en la vida, salvo por el desarrollo de circunstancias que puedan implicar cambios importantes como síndrome nefrótico o tratamiento para reducción Lp (a). No se recomienda la conversión entre unidades nmol/L a mg/dL, o viceversa, ya que todos los factores de conversión dependen intrínsecamente de las isoformas (Material Suplementario Tabla S7).

### ¿Debemos valorar la inflamación en el paciente con arteriosclerosis?

Los procesos inflamatorios crónicos se asocian con un aumento del riesgo cardiovascular independiente del riesgo atribuible por los factores convencionales [56]. La proteína C reactiva de alta sensibilidad es el parámetro analítico que más se ha empleado como marcador de inflamación de baja intensidad. Presenta alta variabilidad y no existe un consenso definido de los valores que deben considerarse como ‘elevados’ para la estimación del riesgo vascular [19].

### Innovación en el diagnóstico de dislipidemias: parámetros necesarios para resolución de una e−consulta

Con el fin de poder realizar una adecuada resolución de la consulta de manera ágil y eficaz, los parámetros mínimos recomendados a incluir en e–consultas para el diagnóstico de dislipidemias se recogen en la Tabla 4.

**Tabla 4:** Datos de referencia necesarios para evaluación riesgo cardiovascular en e–consulta.

Edad, sexo, IMC y perímetro de cintura/cadera del paciente. Breve resumen de antecedentes familiares y personales. Factores de riesgo: Hábito tabáquico, consumo de alcohol, ERC. Breve resumen de historia lipídica y tratamientos hipolipemiantes previos. Tratamiento completo del paciente. Posibles efectos secundarios del tratamiento con fármacos hipolipemiantes. Perfil lipídico básico actual Colesterol total, c-LDL, c-HDL, c-No HDL, TG. Problema actual. Existencia de estudios genéticos personales o familiares previos. En caso de sospecha de Hipercolesterolemia familiar; puntuación de las Red de Clínicas de Lípidos Holandesas/OMS (DLCN) [47]. En caso de sospecha de Hipertrigliceridemia: Puntuación de Moulin para. diagnóstico de quilomicronemia familiar [57].

## Supplementary Material

Supplementary Material

## Material Suplementario

Este artículo incluye material suplementario (https://doi.org/10.1515/almed-2023-0010).
