# Supplementary material for: Documento de consenso para la determinación e informe del perfil lipídico en laboratorios clínicos españoles: ¿Qué parámetros debe incluir un perfil lipídico básico?
Source: Adv Lab Med. 2023 May 18;4(2):147–56. [Article in Spanish] doi: 10.1515/almed-2023-0010 (PMC10701506; doi:10.1515/almed-2023-0010)
Supplement: Supplementary file 1 — Supplementary Material [file j_almed-2023-0010_suppl_001.docx]

**Material suplementario**

**Anexo 1A.** Causas de hipercolesterolemia secundaria
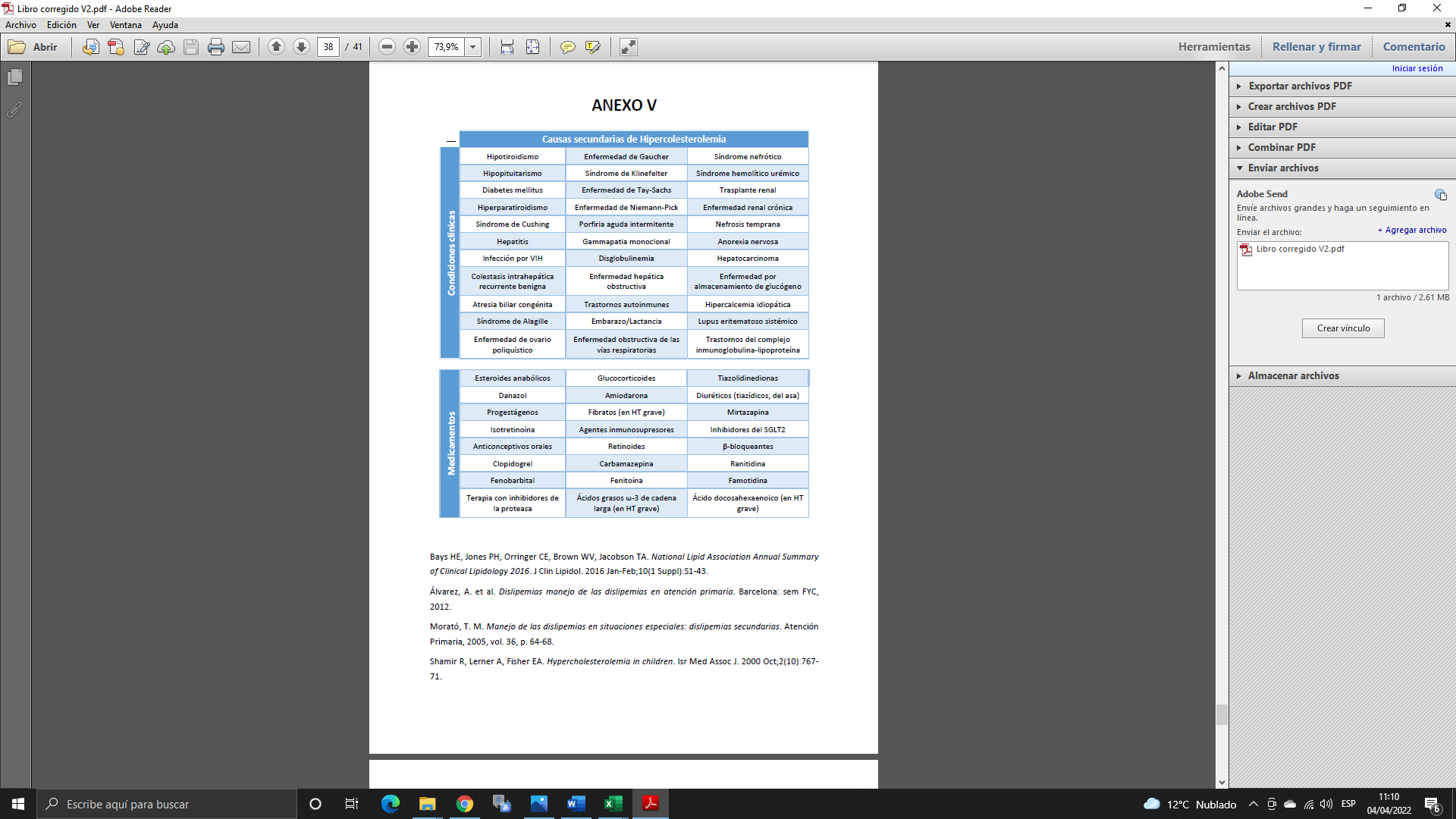


| **Anexo 1B.** Fármacos que pueden causar dislipemias | | | |
| --- | --- | --- | --- |
| **Fármacos** | **Colesterol LDL** | **Trigicéridos** | **Colesterol HDL** |
| ***Cardiovascular /Endocrinología*** | | | |
| Amiodarona | ↑Variable | ↔ | ↔ |
| *β*-Bloqueantes*** | ↔ | ↑10-40% | ↓5-20% |
| Diuréticos del asa | ↑5-10% | ↑5-10% | ↔ |
| Thiazide diuretics (high dose) | ↑5-10% | ↑5-15% | ↔ |
| Inhibidores SGLT2 (cotransportador sodo-glucosa 2) | ↑3-8% | ↔↓ | ↑Variable |
| ***Esteroides*** | | | |
| Estrogenos | ↓7-20% | ↑40% | ↑5-20% |
| Progestágenos | ↑Variable | ↓Variable | ↓15-40% |
| Moduladores selectivos de receptores estrogénicos | ↓10-20% | ↑0-30* | ↔ |
| Danazol | ↑10-40% | ↔ | ↓50% |
| Esteroides anabolizantes | ↑20% | ↔ | ↓20-70% |
| Corticosteroides | ↑Variable | ↑Variable | ↔ |
| ***Antivirales*** | | | |
| Inhibidores de la proteasa | ↑15-30% | ↑15-200% | ↔ |
| Antivirales directos | ↑12-27% | ↔ | ↑14-20% |
| ***Inmunosupresores*** | | | |
| Ciclosporina y tacrólimus | ↑0-50% | ↑0-70% | ↑0-90% |
| Corticosteroides | ↑Variable | ↑Variable | ↔ |
| ***Medicacion con acción Sistema Nervioso Central*** | | | |
| Antipsicóticos de 1ª generación | ↔ | ↑22% | ↓20% |
| Antipsicóticos de 2ª generación | ↔ | ↑20-50% | ↔ |
| Anticomiciales | ↑Variable | ↔ | ↑Variable |
| ***Otros*** | | | |
| Retinoides | ↑15% | ↑35-100% | ↔** |
| Hormona del crecimiento | ↑10-25% | ↔ | ↔↑7% |
| *Raloxifeno no se asocia con aumento de triglicéridos, pero éstos pueden aumentar hasta un 30% con tamoxifeno  **Datos controvertidos  ***Varía con los fármacos individuales.  Referencia  Herink M, Ito MK. Medication Induced Changes in Lipid and Lipoproteins. In: Feingold KR, Anawalt B, Boyce A, et al., eds. *Endotext*. South Dartmouth (MA): MDText.com, Inc.; May 10, 2018. | | | |

**Anexo 2**

**Variabilidad analítica**

**Anexo 3.** Cocientes TG/ VLDL colesterol basada en niveles de TG y colesterol no HDL (modificación de la ecuación de Friedewald por Martin Hopkins). Adaptado de referencia

|  | | | Colesterol no HDL, mg/dL | | | |  |  |
| --- | --- | --- | --- | --- | --- | --- | --- | --- |
| Triglicéridos, mg/dL | <100 | 100-129 | | 130-159 | 160-189 | 190-219 | | ≥220 |
| 7-49 | 3,5 | 3,4 | | 3,3 | 3,3 | 3,2 | | 3,1 |
| 50-56 | 4,0 | 3,9 | | 3,7 | 3,6 | 3,6 | | 3,4 |
| 57-61 | 4,3 | 4,1 | | 4,0 | 3,9 | 3,8 | | 3,6 |
| 62-66 | 4,5 | 4,3 | | 4,1 | 4,0 | 3,9 | | 3,9 |
| 67-71 | 4,7 | 4,4 | | 4,3 | 4,2 | 4,1 | | 3,9 |
| 72-75 | 4,8 | 4,6 | | 4,4 | 4,2 | 4,2 | | 4,1 |
| 76-79 | 4,9 | 4,6 | | 4,5 | 4,3 | 4,3 | | 4,2 |
| 80-83 | 5,0 | 4,8 | | 4,6 | 4,4 | 4,3 | | 4,2 |
| 84-87 | 5,1 | 4,8 | | 4,6 | 4,5 | 4,4 | | 4,3 |
| 88-92 | 5,2 | 4,9 | | 4,7 | 4,6 | 4,4 | | 4,3 |
| 93-96 | 5,3 | 5,0 | | 4,8 | 4,7 | 4,5 | | 4,4 |
| 97-100 | 5,4 | 5,1 | | 4,8 | 4,7 | 4,5 | | 4,3 |
| 101-105 | 5,5 | 5,2 | | 5,0 | 4,7 | 4,6 | | 4,5 |
| 106-110 | 5,6 | 5,3 | | 5,0 | 4,8 | 4,6 | | 4,5 |
| 111-115 | 5,7 | 5,4 | | 5,1 | 4,9 | 4,7 | | 4,5 |
| 116-120 | 5,8 | 5,5 | | 5,2 | 5,0 | 4,8 | | 4,6 |
| 121-126 | 6,0 | 5,5 | | 5,3 | 5,0 | 4,8 | | 4,6 |
| 127-132 | 6,1 | 5,7 | | 5,3 | 5,1 | 4,9 | | 4,7 |
| 133-138 | 6,2 | 5,8 | | 5,4 | 5,2 | 5,0 | | 4,7 |
| 139-146 | 6,3 | 5,9 | | 5,6 | 5,3 | 5,0 | | 4,8 |
| 147-154 | 6,5 | 6,0 | | 5,7 | 5,4 | 5,1 | | 4,8 |
| 155-163 | 6,7 | 6,2 | | 5,8 | 5,4 | 5,2 | | 4,9 |
| 164-173 | 6,8 | 6,3 | | 5,9 | 5,5 | 5,3 | | 5,0 |
| 174-185 | 7,0 | 6,5 | | 6,0 | 5,7 | 5,4 | | 5,1 |
| 186-201 | 7,3 | 6,7 | | 6,2 | 5,8 | 5,5 | | 5,2 |
| 202-220 | 7,6 | 6,9 | | 6,4 | 6,0 | 5,6 | | 5,3 |
| 221-247 | 8,0 | 7,2 | | 6,6 | 6,2 | 5,9 | | 5,4 |
| 248-292 | 8,5 | 7,6 | | 7,0 | 6,5 | 6,1 | | 5,6 |
| 293-399 | 9,5 | 8,3 | | 7,5 | 7,0 | 6,5 | | 5,9 |
| ≥ 400 | 11,9 | 10,0 | | 8,8 | 8,1 | 7,5 | | 6,7 |

**Anexo 4.** Criterios de la Red de Clínicas de Lípidos Holandesas para el Diagnóstico de Hipercolesterolemia familiar


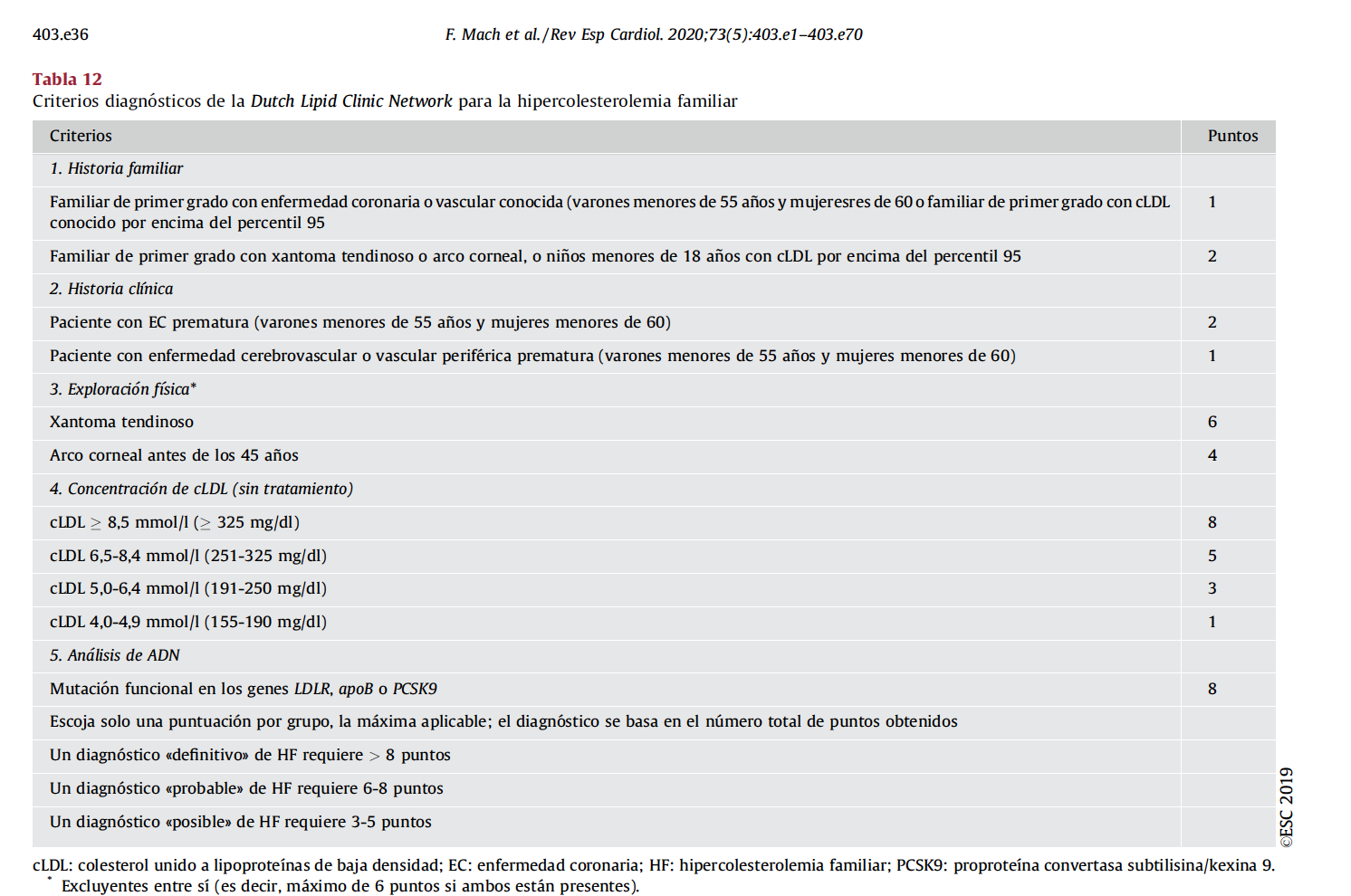


| **Anexo 5**. Puntuación de Moulin para el diagnóstico práctico del Síndrome de Quilomicronemia Familiar | |
| --- | --- |
| **Fase de reclutamiento**  Hipertrigliceridemia severa en ayunas (>10 mmol/L or 885 mg/dL) | |
| **Pre-selección de pacientes** (fuera de un episodio agudo) | |
| 1. TG en ayunas >10 mmol/L durante 3 análisis de sangre consecutivos (+5)^a^    1. TG en ayunas >20 mmol/L al menos una vez (+1) 2. TG <2 mmol/L en determinaciones previas (-5) 3. Ausencia de causas de hiperlipemia secundaria ^b^ (excepto embarazo^c^ y tratamiento con etinil estradiol) (+2) 4. Historia de pancreatitis (+1) 5. Dolor abdominal recurrente inexplicable (+1) 6. No historia de hiperlipemia familiar combinada (+1) 7. Pobre respuesta a tratamiento hipolipemiante (disminución de TG <20%) 8. Edad de Inicio de los síntomas:    1. <40 años (+1)    2. <20 años (+2)    3. <10 años (+3) | Puntuación para Síndrome de quilomicronemia familiar:  ≥10: Muy probable  ≤9: FCS Improbable  ≤8: FCS Muy improbable |
| Los Números entre paréntesis indican la ponderación otorgada a cada item.  La puntuación para el diagnóstico del síndrome de quilomicronemia familiar es la suma de todos los elementos presentes.  ^a^ medidas al menos con un mes de diferencia.  ^b^ Incluyendo alcohol, diabetes, síndrome metabólico, hipotiroidismo, corticoterapia y fármacos  ^c^ Si el diagnóstico se realiza durante el embarazo es necesaria una segunda evaluación para confirmar el diagnóstico posparto. | |

**Anexo 6**. Algoritmo simplificado para el diagnóstico de disbetalipoproteinemia ^45^


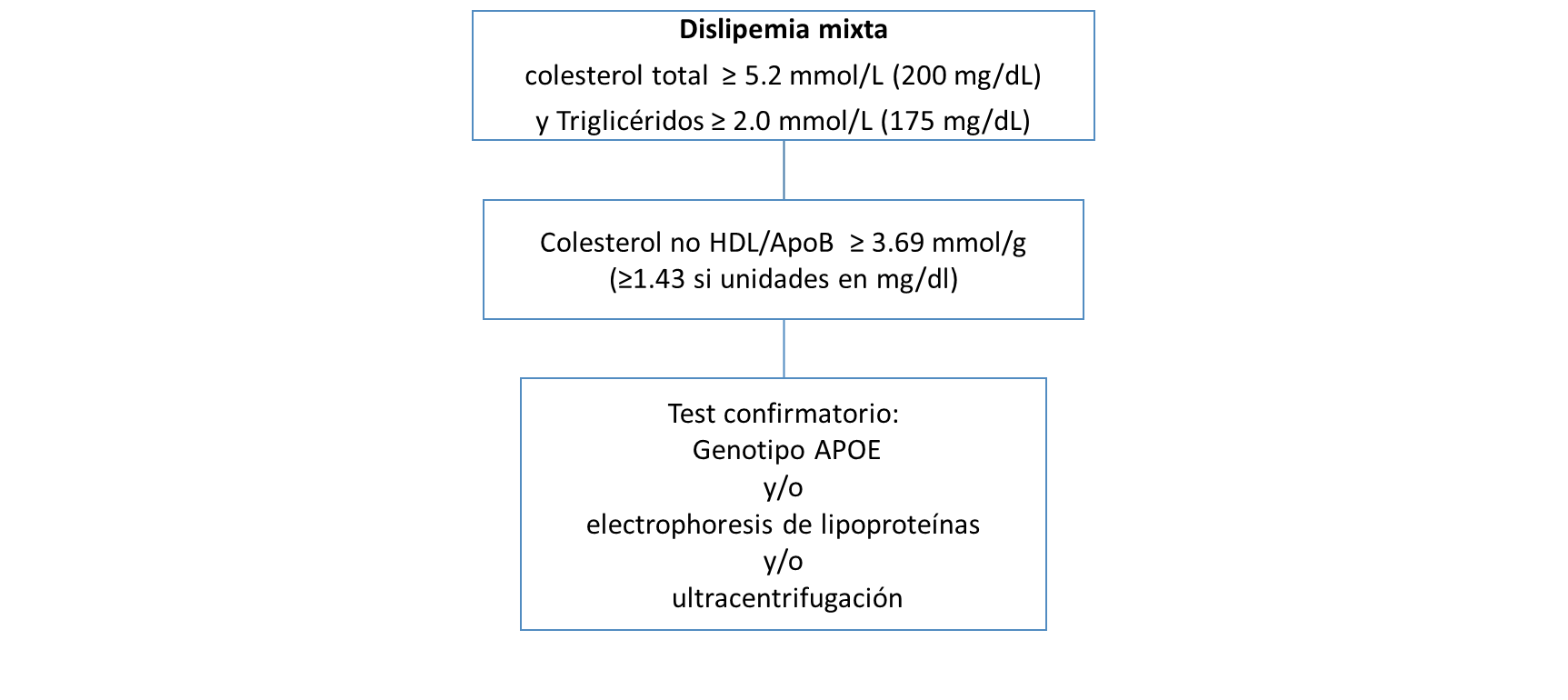


**Anexo 7**:

Controversias en unidades de determinación Lp(a):

En la práctica clínica, la determinación de los niveles de Lp(a) se lleva a cabo, generalmente, mediante inmunoensayos que utilizan anticuerpos policlonales frente a la apo(a). Los resultados se suelen expresar como masa total de la partícula de Lp(a) en (mg/dL), que incluye tanto la masa de apo(a) como la del resto de componentes de la partícula (apoB, colesterol, ésteres de colesterol, fosfolípidos, triglicéridos y carbohidratos) o como concentración de molar de apo(a) en nmoL/L. Desde el punto de vista metrológico, la expresión de resultados como masa total (mg/dL) cuenta con importantes limitaciones debido a la gran variabilidad que presenta la Lp(a) en su composición y a la falta de un material de referencia al que referir los calibradores. Por el contrario, la expresión de resultados en nmol/L de apo(a) refleja de una manera mas precisa la concentración plasmática de partículas de Lp(a) y permite además, la comparación entre diferentes métodos al contar con un material de referencia (OMS/IFCC SRM-2B) al que se pueden referenciar los calibradores.

No se recomienda la conversión entre unidades nmol/L a mg/dL, o viceversa, ya que todos los factores de conversión dependen intrínsecamente de las isoformas
